# Catechol‐O‐methyltransferase Val158Met polymorphism associates with affect and cortisol levels in women

**DOI:** 10.1002/brb3.883

**Published:** 2018-01-18

**Authors:** Lauren D. Hill, Margaret S. Lorenzetti, Sarah M. Lyle, Ana I. Fins, Aurélien Tartar, Jaime L. Tartar

**Affiliations:** ^1^ Department of Psychology and Neuroscience Nova Southeastern University Fort Lauderdale FL USA; ^2^ Department of Clinical and School Psychology Nova Southeastern University Fort Lauderdale FL USA; ^3^ Department of Biological Sciences Nova Southeastern University Fort Lauderdale FL USA

**Keywords:** affect, catechol‐O‐methyltransferase, cortisol, dopamine, emotion, women

## Abstract

**Introduction:**

We tested the extent to which the catechol‐O‐methyltransferase (COMT) Val158Met polymorphism is associated with affective state and evening cortisol levels. We limited our study to women as previous research suggests that the link between COMT genotype and psychological health is entangled by sex differences.

**Materials and Methods:**

The participants were assessed on measures of anxiety, mood disturbance, depressive symptomatology, and perceived stress. We also evaluated participants on a quality of life measures that included two emotion domains and two physical domains (physical health and environment).

**Results:**

We found that under normal (nonstress) conditions, the COMT A allele (Met carriers, higher dopamine) associates with healthier affect and lower afternoon cortisol levels in women. These effects were limited to affective measures and not to physical or environmental quality of life.

**Conclusions:**

These findings help to shed light on the complex nature of COMT and emotion, and suggest that both sex and task condition (stress vs. nonstress) should be considered when examining the relationship between COMT genotype and emotion.

## 1 INTRODUCTION

Understanding the genetic factors that affect neurotransmitter variations can help explain the multifaceted neurobiological processes that underlie emotion processing as well as individual differences in susceptibility to mood disorders. A functional single‐nucleotide polymorphism (SNP) in the catechol‐O‐methyltransferase (COMT) gene (rs4680) holds great promise as a gene variant that can predict individual differences in emotion processing. The COMT enzyme works to catabolize catecholamines in the central and peripheral nervous systems. The COMT SNP is characterized by a substitution of methionine (Met) in place of valine (Val) at codon 158 (Val158Met), which results in a twofold to fourfold decrease in the activity of the COMT enzyme (Lotta et al., 1995; Männistö & Kaakkola, 1999). In the prefrontal cortex (PFC), the COMT enzyme plays a particularly critical role in the breakdown of dopamine (DA) as the DA transporter (DAT) has low expression in PFC synapses (Karoum, Chrapusta, & Egan, 1994; Lewis et al., 2001; Matsumoto et al., 2003). The functional effects of the COMT SNP on DA neurotransmission in the PFC have been documented with the Met/Met homozygote mice showing higher DA levels (Akil et al., 2003).

The COMT allele status has also been shown to *functionally* alter DA activity in the PFC wherein COMT Met (low‐activity; high dopamine) allele carriers outperform Val (high‐activity; low dopamine) allele carriers on a variety of cognitive tasks (Bruder et al., 2005; Diaz‐Asper et al., 2008; Egan et al., 2001; Goldberg et al., 2003). Interestingly, this relationship between genotype and cognitive performance appears to reverse under stressful conditions. Stress increases PFC DA levels, and Met allele carriers (with higher DA) show performance deficits relative to Val allele carriers. This pattern reflects the inverted U‐shaped function of DA activity where too little (Val allele) or too much (Met allele carriers under stress) DA is associated with poor cognitive performance (Goldman‐Rakic, Muly, & Williams, 2000).

In agreement with findings in the cognitive literature, research suggests that after exposure to stress, the development of mood and anxiety disorders associates with the Met allele (Mandelli et al., 2007). However, in the absence of stressful conditions, poor emotion processing has been alternatively associated with Met allele carriers in some studies (Drabant et al., 2006; Enoch, Waheed, Harris, Albaugh, & Goldman, 2009; Woo, Yoon, & Yu, 2002) and with Val allele carriers in other studies (Hamilton et al., 2002; Ohara, Nagai, Suzuki, & Ohara, 1998; Shulman, Griffiths, & Diewold, 1978). The Val allele advantage for emotional and stress resiliency is referred to as the COMT “warrior/worrier” model (Goldman, Oroszi, & Ducci, 2005).

The link between COMT genotype and psychological health appears further entangled by potential sex differences. The discrepancy between men and women on psychological measures has been comprehensively established (Pavlova, 2016; Zagni, Simoni, & Colombo, 2016) and may be related to sex differences in the role of catecholamine regulation in anxiety and mood disorders (Domschke et al., 2004). Compared to men, women have significantly more DA cells within the mesocortical pathway, a major dopaminergic pathway projecting to PFC (50% vs. 30%, respectively) (Kritzer & Creutz, 2008; Swanson, 1982). Usually, these sexual dimorphisms are attributed to the influence of sex hormones and actions of sex chromosome genes (Harrison & Tunbridge, 2008). It is possible that estrogen mediates the sexually dimorphic nature of DA activity in the PFC as there is estrogen receptor (ER)β expression in DA neurons that project to the PFC (Creutz & Kritzer, 2002). It is likely that in nonstressed women, the Met allele associates with healthy emotion processing. Accordingly, a recent study demonstrated that women Met homozygotes were less sensitive to negative faces than women Val homozygotes (Weiss et al., 2007). Moreover, the Val allele is associated with panic disorder in females but not males (Domschke et al., 2004; Hamilton et al., 2002).

The goal of this study was to address the uncertainty surrounding the extent to which the COMT Val158Met polymorphism is associated with affective processing in women. To that end, we examined the association between COMT allele type and measures that spanned various facets of affective states (Ekkekakis, 2013) including depressive symptomology, perceived stress, and mood disturbances in women. We also administered a quality of life assessment which teases apart emotion domains (psychological and social) from physical domains (physical health and environment). Further, we measured cortisol levels as previous work proposed an association between the COMT Val158Met polymorphism, cortisol secretion, and emotion. We hypothesized that, relative to Val allele carriers, Met allele carriers would exhibit signs of better emotion processing across measures of emotion, mood, and affect as well as lower evening cortisol levels.

## 2 MATERIALS AND METHODS

### 2.1 Participants

Seventy‐eight participants (mean age = 21.12, *SD* = 5.17) were recruited through flyers posted in public buildings and through the NSU participant pool. Exclusion criteria during study enrollment included being younger than 18 years of age or over 50 years of age, having a positive history of mental illness, taking medication for sleep, taking psychotropic medication, or a diagnosis of a sleep disorder. Self‐reported race/ethnicities were as follows: 59 White/Caucasian, nine Black/African American, six Asian, three Multiracial, and one unidentified; 17 participants self‐reported to be Hispanic. All participants were compensated with a $10 store gift card. The testing procedures were carried out according to a protocol reviewed and approved by the Nova Southeastern University Institutional Review Board.

### 2.2 Procedure

All participants signed a written consent form, provided two saliva samples (one for DNA extraction and one for cortisol quantification), and completed a series of psychological instruments to measure affective states and quality of life. Testing occurred between 6:00 and 8:00 p.m.—a time when cortisol levels are naturally low. Participants provided saliva samples for cortisol quantification and DNA extraction via passive drool though a straw into 1.5 ml microcentrifuge tubes after they filled out the inventories.

### 2.3 Emotion processing inventories

#### 2.3.1 State‐Trait Anxiety Inventory (STAI‐Y)

State and trait anxiety were measured using the STAI‐Y (Spielberger, Gorsuch, Lushene, Vagg, & Jacobs, 1983). The Trait and State scales each consist of 20 items. This instrument has been used extensively in research and clinical practice. Spielberger et al. (1983) report internal consistency coefficients for young adult females to be 0.93 for State anxiety and 0.92 for Trait anxiety. Test–retest reliability coefficients range between 0.65 and 0.75 (Spielberger et al., 1983). Moreover, it has been validated as an accurate measure of anxiety in adults (Okun, Stein, Bauman, & Silver, 1996) and convergent and discriminant validation has been exhibited when compared with other measures (Grös, Antony, Simms, & McCabe, 2007).

#### 2.3.2 Profile of Mood States (POMS)

The POMS was utilized in this study to measure acute mood (“How do you feel right now”) and ongoing mood (“How have you been feeling during the past week, including today”) (McNair, Lorr, & Droppleman, 1971). It consists of 65 items that tap six scales assessing anger–hostility, confusion–bewilderment, depression–dejection, fatigue–inertia, tension–anxiety, and vigor–activity in addition to a composite score of total mood disturbance. Internal consistencies vary from 0.84 for the confusion–bewilderment scale to 0.95 for the depression–dejection scale, while test–retest reliabilities range from 0.65 for vigor to 0.74 for depression (McNair, Lorr, & Droppleman, 1992). McNair et al. (1992) also provided supportive evidence for the instrument's criterion‐related validity.

#### 2.3.3 The Center for Epidemiologic Studies Depression Scale (CES‐D)

The CES‐D was employed to measure depressive symptomatology (Radloff, 1977). Unlike other depression scales that focus on clinical populations, the CES‐D was created to be utilized with a general (nonclinical) population. Twenty items are rated on a 4‐point Likert scale. In a college sample, Cronbach's α was found to be 0.87 (Radloff, 1977) reported moderate test–retest correlations ranging from 0.32 (one‐year retest interval) to 0.68 (four‐month interval). The instrument also accurately discriminates between patient and nonpatient groups (Radloff, 1991).

#### 2.3.4 Perceived Stress Scale (PSS)

The 10‐item PSS was applied to measure current stress levels in the participants and as a complement to cortisol measures (Cohen & Williamson, 1988). It exhibits acceptable internal consistency with Cronbach's α ranging from 0.78 to 0.91 (Cohen et al., 2012). Construct validity has been demonstrated via the relationships between the instrument, various measures of stress, and sources of stress, as well as measures of health and health behaviors (Cohen et al., 2012).

### 2.4 Quality of life measures

#### 2.4.1 World Health Organization Quality of Life (WHOQOL‐BREF)

The WHOQOL‐BREF instrument was used to investigate different aspects associated with quality of life. As this instrument assesses four domains: physical health (WHOQOL 1), psychological health (WHOQOL 2), social relationships (WHOQOL 3), and environment (WHOQOL 4), we were able to isolate emotion components (psychological health and social relationships) from physical factors and environmental factors (Skevington & O'Connell, 2004). The instrument is an abbreviated version of the WHOQOL‐100. Cronbach's α coefficients range from 0.66 for domain 3 to 0.84 for domain 1, while two to eight‐week test–retest reliabilities for the domains ranged from 0.66 for domain 1 to 0.87 for domain 4 (Group, 1998).

### 2.5 Biomarkers

#### 2.5.1 Cortisol

Saliva samples were run in duplicate and quantified via a human cortisol enzyme immunoassay (EIA) kit per the manufacturer's instructions (Salimetrics LLC, USA). The samples were immediately read in a BioTek ELx800 plate reader (BioTek Instruments, Inc., USA) at 450 nm with a correction at 630 nm. All samples were within the detection ranges indicated in the cortisol immunoassay kit, and the variations of sample readings were within the expected limits. Final concentrations for the biomarkers were generated by interpolation from the standard curve in μg/dl.

#### 2.5.2 Genotyping

Genomic DNA was extracted in a QIAcube instrument following the manufacturer's standard protocol for saliva nucleic acid extraction (QIAGEN, Valencia, CA, USA). After isolation, allelic discrimination for the COMT gene was determined via real‐time polymerase chain reaction (PCR) using a TaqMan SNP genotyping assay using fluorogenic probes (Applied Biosystems, CA, USA). Thermal cycling was performed on StepOne Real‐Time PCR system (Applied Biosystems). The amplification mix contained the following ingredients: 12.5 μl of PCR master mix (QIAGEN), 1.25 μl of TaqMan 20× working stock, 10.25 μl of RNase‐ and DNase‐free water (Sigma), and 1.0 μl of sample DNA, in a total volume of 25 μl per single‐tube reaction. The PCR conditions were 95°C for 10 min followed by 50 repeated cycles of 92°C for 15 s and 60°C for 90 s. Genotypes were determined automatically via the StepOne software (Applied Biosystems) based on the fluorescence signals. Samples were run in duplicate and in the case of a call discrepancy, samples were rerun.

### 2.6 Statistical analyses

We conducted a series of independent samples *t* tests to assess the relationship between COMT genotype and emotion processing, and COMT and cortisol. The distribution of allele frequencies was determined by the Hardy–Weinberg Exact (HWE) test, and the association of allele status was analyzed using the chi‐square test. All calculations were conducted using an SPSS statistical package (version 19, SPSS inc., IBM). All reported *p*‐values are two‐tailed with a priori significance level of *p* < .05.

## 3 RESULTS

### 3.1 Genotype frequency

Catechol‐O‐methyltransferase genotype frequencies were as follows: 22% AA, 50% AG, and 28% GG. The HWE test showed that χ^2^ = 1.24, *p* > .05, suggesting that the population is consistent with Hardy–Weinberg Equilibrium, and confirming that the allele types were randomly sampled. In order to examine the hypothesized benefit of the Met (A) allele, we collapsed across genotypes containing the Met allele. The AA homozygotes (Met/Met) and the AG heterozygotes (Met/‐) (*n* = 54) were compared to the GG homozygotes (Val/Val) (*n* = 24).

### 3.2 Association between COMT and emotion processing measures

**Figure 1:**
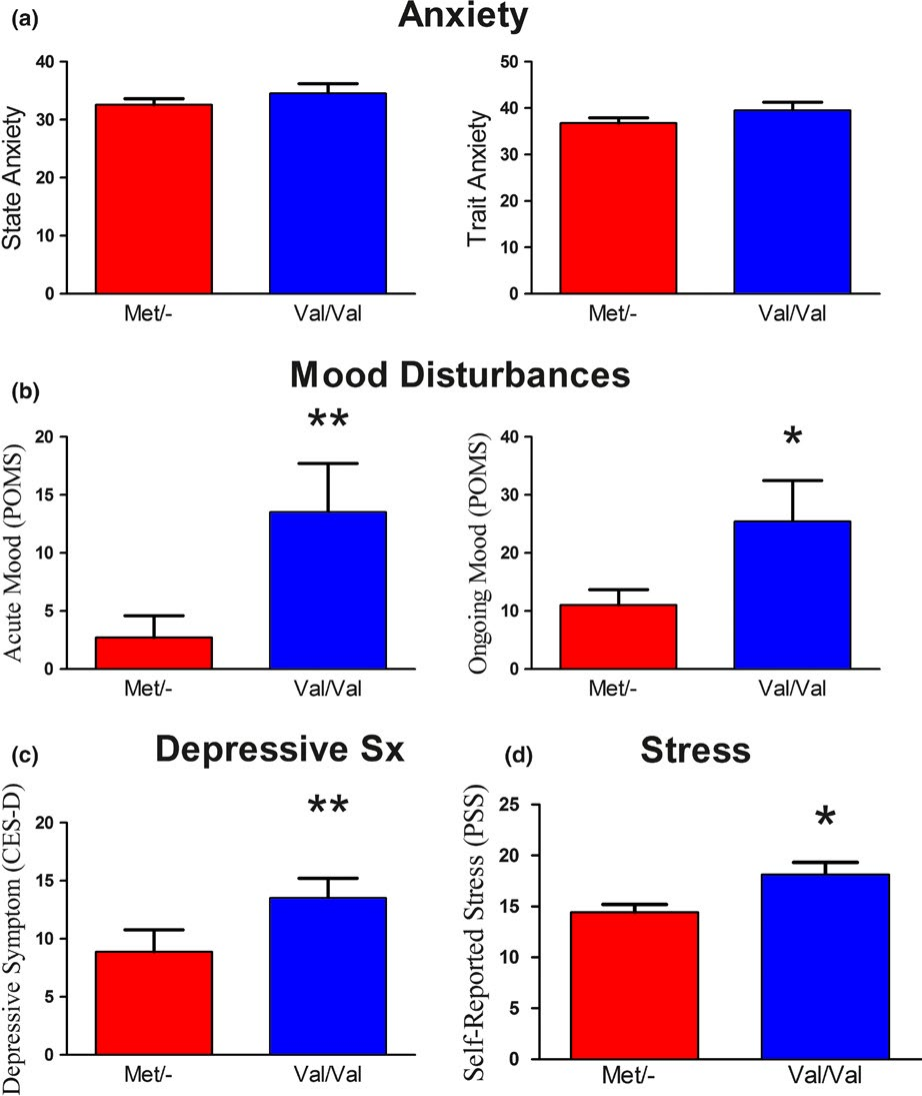
Results on affect inventories as a function of COMT genotype. (a) No significant difference between Met allele carriers and the Val/Val group on STAI, state anxiety *t*(76) = −1.00, *p* = .32, trait anxiety *t*(76) = −1.33, *p* = .19. (b) Significantly lower scores on acute mood disturbance *t*(76) = −2.70, *p* = .009, and ongoing mood disturbance *t*(76) = −2.34, *p* = .02 for Met allele carriers measured via the POMS. (c) A significant increase in depressive symptomatology *t*(76) = −2.95, *p* = .004, for Val/Val genotypes (CES‐D). (d) Perceived stress (PSS) was significantly lower in Met allele carriers than the Val/Val group *t*(76) = −2.63, *p* = .01. COMT, catechol‐O‐methyltransferase; POMS, Profile of Mood States

Means and standard deviations for the emotion processing inventories as a function of COMT genotype are listed in Table 1 and are depicted in Figure 1. The STAI did not show a significant difference between the A (Met) allele carriers and GG (Val) homozygotes for either state anxiety *t*(76) = −1.00, *p* = .32 or trait anxiety *t*(76) = −1.33, *p* = .19. Met allele carriers showed lower mood disturbance scores compared to Val homozygotes for the acute mood disturbance measure *t*(76) = −2.70, *p* = .009 as well as the ongoing mood disturbance measure *t*(76) = −2.34, *p* = .02 of the POMS. The CES‐D also showed an emotion advantage for the A allele carriers relative to the GG homozygotes on depressive symptomatology *t*(76) = −2.95, *p* = .004. Perceived stress (PSS) was also significantly lower in Met allele carriers than the Val/Val group *t*(76) = −2.63, *p* = .01.

**Table 1:** COMT genotypes and emotion measures

Measure	A/‐		A/G		*t*	*p*
	*M*	*SD*	*M*	*SD*		
STAI state	32.60	7.65	34.54	9.37	−1.00	.32
STAI trait	36.79	8.00	39.50	8.93	−1.33	.19
POMS acute	2.75	14.00	13.52	20.05	−2.70	**.01**
POMS ongoing	11.06	19.03	25.44	33.97	−2.34	**.02**
CES‐D	8.89	5.97	13.83	8.21	−2.45	**<.01**
PSS	14.43	5.68	18.13	5.81	−2.64	**.01**
WHO physical	23.11	3.13	22.17	2.97	1.25	.22
WHO psychological	22.75	2.99	20.83	3.31	2.53	**.01**
WHO social	11.43	2.44	10.08	2.83	2.14	**.04**
WHO environment	32.53	4.94	30.04	4.98	2.04	.05
Cortisol (μg/dl)	0.19	0.11	0.28	0.23	−0.20	**.04**

*M*, mean; *SD*, standard deviation; significant *p* values are emboldened.

STAI, State‐Trait Anxiety Inventory; POMS, Profile of Mood States; CES‐D, The Center for Epidemiologic Studies Depression Scale; PSS, Perceived Stress Scale; WHO, World Health Organization Quality of Life measures; COMT; catechol‐O‐methyltransferase.

### 3.3 Quality of life measures

**Figure 2:**
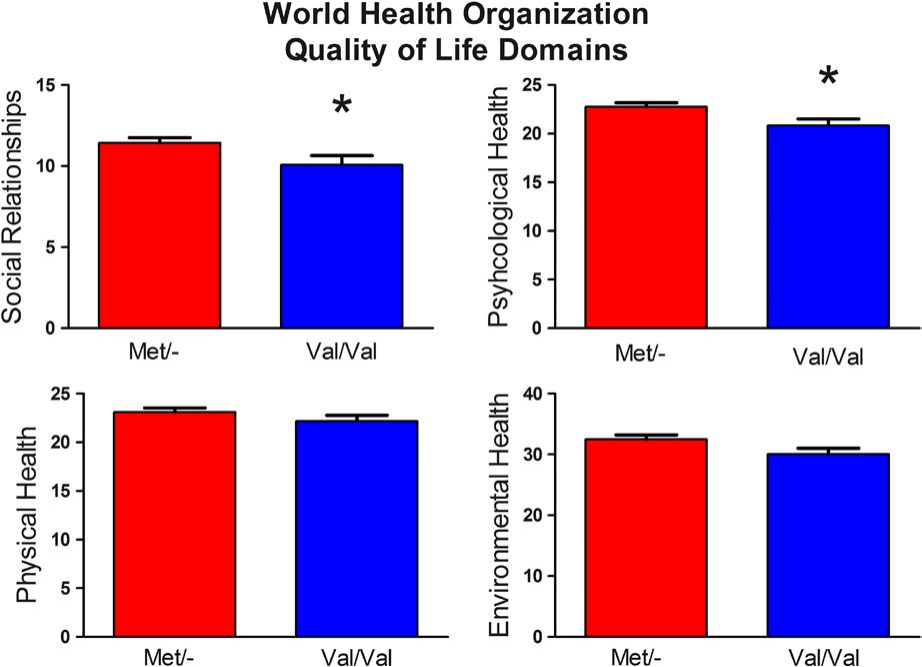
Four domains of the WHOQOL‐BREF inventory. Met allele carriers reported better social relationships *t*(76) = 2.14, *p* = .04 and psychological health *t*(76) = −2.53, *p* = .01 compared to Val/Val genotypes. No significant group difference on the measure of physical health *t*(76) = 1.25, *p* = .22 or environmental health *t*(76) = −2.041, *p* = .05

On the two emotion domains of the WHOQOL‐BREF inventory, Met allele carriers reported better psychological health *t*(76) = −2.53, *p* = .01 and social relationships *t*(76) = 2.14, *p* = .04 compared to Val/Val allele carriers. Interestingly, there was a marginally significant difference on the measure of environmental health *t*(76) = −2.041, *p* = .05. There was not a significant group difference on the measure of physical health *t*(76) = 1.25, *p* = .22 (Figure 2).

### 3.4 Cortisol

**Figure 3:**
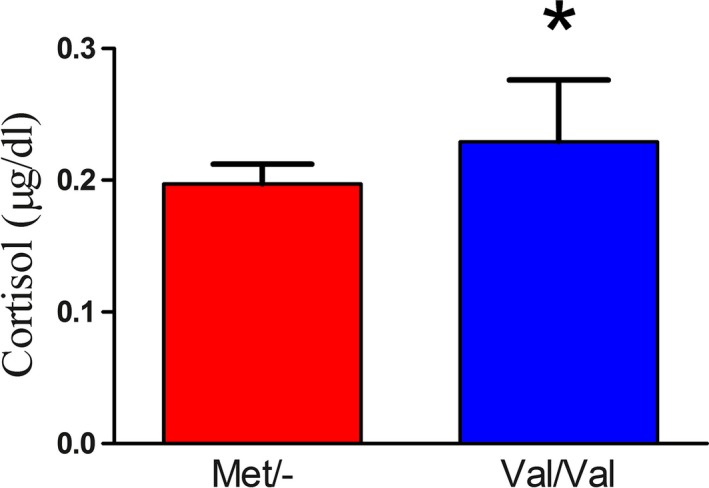
Differences in cortisol levels for Met allele carriers and Val/Val allele carriers. Cortisol levels were significantly lower in the Met/‐ group relative to the Val/Val group *t*(76) = −2.63, *p* = .01

Cortisol was measured as previous work suggested relationship between cortisol and COMT and that stress might alter the effect of the COMT genotype on performance measures. In agreement with our overall findings that Met allele carriers score higher on measures of emotion processing, cortisol levels were significantly lower in the Met/‐ group relative to the Val/Val group *t*(76) = −2.63, *p* = .01 (see Table 1 and Figure 3).

## 4 DISCUSSION

Our findings show that relative to Val homozygotes, COMT Met allele carriers report better affective states across a variety of validated self‐report measures. In addition, compared to Val homozygotes, women Met allele carriers have significantly lower cortisol levels.

Previous reports on the relationship between the COMT genotype and emotion are conflicting. There is general support for the “warrior/worrier” model of COMT (Goldman et al., 2005) which posits that the Val (warrior) allele confers an advantage for emotional resiliency in threatening environments, while the Met (worrier) allele confers an advantage in complex memory and attention tasks. However, a body of research points to the notion that the relationship between COMT allele status and emotion is perhaps more nuanced than the dichotomous “warrior/worrier” model. Under some experimental conditions, the Met allele appears to offer an advantage in emotion processing over the Val allele. For example, on a task that involves selecting and manipulating self‐generated thoughts, the Met homozygotes outperform Val carriers (Kilford, Dumontheil, Wood, & Blakemore, 2014). The general association of emotional resiliency with the Met allele has also been observed in a sample of patients with schizophrenia. Relative to Val/Val's, Met/Met schizophrenic homozygotes showed enhanced activation in brain areas related to cognitive control of emotion and lower ratings of distress during an emotional task (Poletti et al., 2013).

Observed behavioral effects may also be sensitive to the sexually dimorphic nature of COMT activity in the PFC (Creutz & Kritzer, 2002; Gogos et al., 1998; Kritzer & Creutz, 2008; Swanson, 1982). These data agree with previous findings that the Val allele associates panic disorder in women (Domschke et al., 2004; Hamilton et al., 2002). In further agreement, an additional study composed predominantly of female participants found that, relative to Val homozygotes, Met allele carriers had smaller visuocortical activation, lower heart rate, and decreased startle potentiation to aversive stimuli (Gruss, Langaee, & Keil, 2016). Another study compared allele status to behavioral risk taking propensity in adolescents and found risk taking to be higher in females, but not males, who were Met allele carriers. These analyses seem at odds with the Met allele carriers representing a “worrier” phenotype (Amstadter et al., 2012), but support our findings that in women, relative to Val homozygotes, Met allele carriers had better emotional health across a variety of self‐report affective state measures and also had lower cortisol under a nonstress condition.

Given that the Met allele is associated with enhanced DA signaling in the PFC, our findings are theoretically sound and consistent with the inverted U‐shaped curve theory of DA activity (Goldman‐Rakic et al., 2000). Our analysis suggests that, under no stress conditions, women Met allele carriers with high dopamine levels, and low COMT activity, demonstrate increased emotional resiliency. It is possible that higher baseline PFC DA levels result in healthier affective states for the Met allele carriers relative to the Val allele carriers. However, under conditions of increased stress or emotion task engagement, high levels of DA in the PFC may lead to emotion processing deficits and behavioral inflexibility in the Met allele carriers, relative to the Val allele carriers.

The World Health Organization quality of life measurement provides additional support for our findings. Specifically, our results showed that Met allele carriers had a significant advantage in the WHOQOL‐BREF domains associated with emotion (psychological and social quality of life) compared to Val homozygotes. This did not appear to be attributable to an overall quality of life bias, or advantage, for Met allele carriers as the environmental and physical quality of life domains were not significantly different between COMT groups.

We found that cortisol levels were lower in Met allele carriers relative to Val homozygotes—in agreement with the trend of our emotion measures. Of note, cortisol values in the present study represent cortisol under a nonstress condition and at a time of day when cortisol levels are low and stable between participants (Chan & Debono, 2010). Drawing from the trend on affective state measures in the current study, and the inverted U‐shaped curve theory of DA activity (Goldman‐Rakic et al., 2000), it is conceivable that increased stress or emotion task engagement would result in higher cortisol levels in the Met allele carriers relative to the Val homozygotes. In fact, the Met allele has already been shown to associate with higher cortisol levels compared to the Val allele in response to stress (Jabbi et al., 2007; Oswald, McCaul, Choi, Yang, & Wand, 2004). Our finding that Val homozygotes had higher cortisol levels than Met allele carriers, under nonstress conditions, is also consistent with the idea that high afternoon cortisol levels are associated with mood impairments (Christensen et al., 1983, 1985). Our findings might be limited to women, however, as a previous study in men failed to find a relationship between COMT genotype and baseline or poststress cortisol levels. (Alexander et al., 2011).

Our sample consisted of a racially diverse group, which could impact the results of this work. In order to test this possibility, we carried out a genotype by race (White vs. non‐White) two‐way ANOVA follow‐up analyses on our variables. We found a significant genotype by race interaction for cortisol (*p* = .02) and the WHO psychological health subdomain (*p* = .03). In both of these measures, the non‐White, GG group were driving the interaction with poorer outcomes (higher cortisol and lower psychological health). However, it is important to note that this study did not aim to investigate racial differences in these measures. Accordingly, future work, with balanced sample sizes and detailed demographics, should further investigate the possibility of racial differences in the influence of COMT on emotion measures.

Due to concerns about potential sex differences combined with previous work showing that the COMT genotype can affect performance under stress, our study was limited to women in a nonstress condition. Therefore, the fact that we only tested women in our study limits the generalizability of the results to a larger population. Yet, these findings provide insights into the potentially sexually dimorphic effects of COMT activity on emotion. These results are meaningful given that women have significantly more DA cells in the mesocortical pathway, and men have 17% higher COMT activity in the PFC (lower dopamine) (Kritzer & Creutz, 2008; Swanson, 1982). Relatedly, our participant sample was comparatively homogenous on numerous demographic variables (young, college‐educated, healthy women), further constricting the generalizability of our results. Of note, however, is that our sample was relatively diverse in terms of race and ethnicity. An additional limitation to the present study was that we did not test the extent to which COMT related to affective measures and cortisol under stress. We are currently carrying out a follow‐up study in a demographically similar group of women to address this question.

In conclusion, results from this study suggest that under normal (nonstress) conditions, the COMT A allele (Met carriers) associates with healthier affective states and lower afternoon cortisol levels in women. These findings help shed light on the complex nature of COMT and emotion, and suggest that both sex and task condition should be considered when examining the relationship between COMT genotype and emotion.

## CONFLICT OF INTEREST

The authors declare that they have no competing or conflict of interest.
